# Supplementary material for: Synchronized LFP rhythmicity in the social brain reflects the context of social encounters
Source: Commun Biol. 2024 Jan 2;7:2. doi: 10.1038/s42003-023-05728-8 (PMC10761981; doi:10.1038/s42003-023-05728-8)
Supplement: Supplementary file 2 — Supplementary Information [file 42003_2023_5728_MOESM2_ESM.pdf]

Supplementary figures

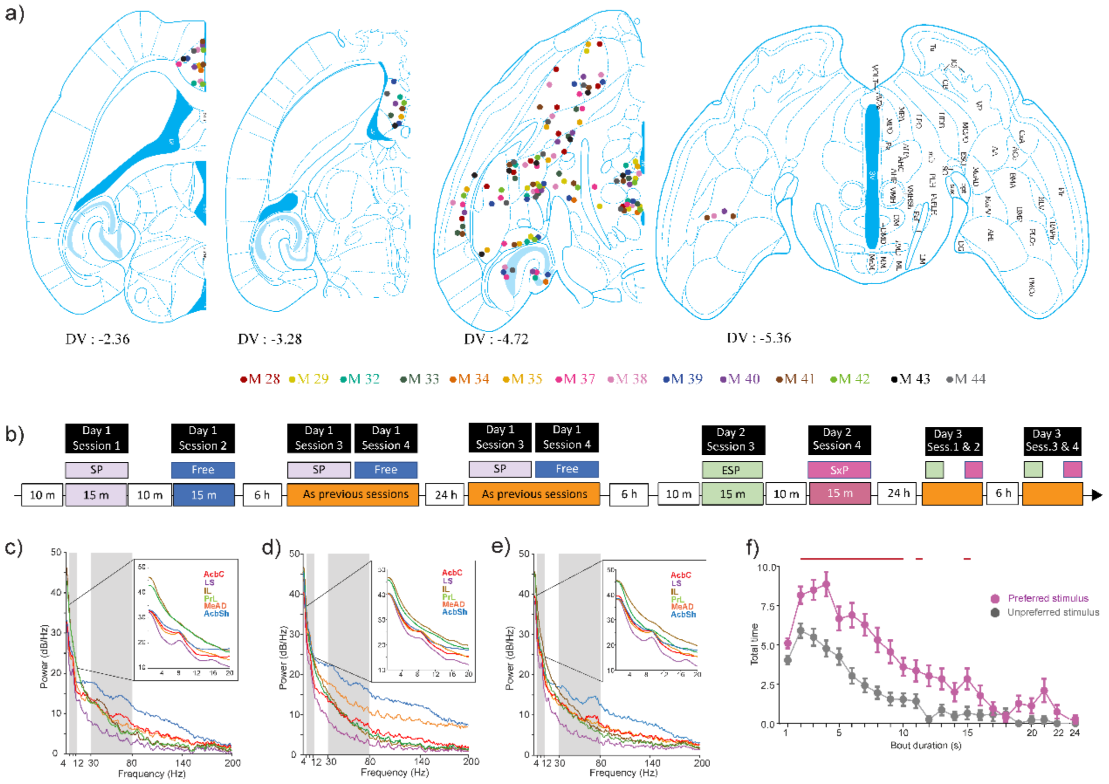

Supplementary Figure 1: Locations of all electrode tips and PSD profiles from individual sessions

a) The locations of all electrode tips, as verified *post mortem*, color-coded for all subject animals (M28-44) on brain atlas pictures. The color code is presented below.

b) Timeline of all sessions conducted by each subject.

c) Power spectral density (PSD) plots of LFP signals recorded in the MeAD, IL, PrL, CA1, AcbC, and AcbSh during the encounter period of a single SP task sessions. The inset shows the PSD plots for 0-20 Hz band at higher resolution.

d) As in **c**, for a single EsP session conducted by the same mouse.

e) As in **c**, for a single SxP session conducted by the same mouse.

f) Histograms of the mean (±SEM) total time devoted by the subject to investigate the preferred (pink) or less-preferred (grey) stimulus across all sessions and tasks, plotted against the bout duration. The horizontal bar above represents the bout durations where a significant difference between the two stimuli was observed. Note that this difference starts at bouts which are longer than 2 s. (2-way ANOVA. Stimulus X TimeBins, Stimulus $F\ (1, 86) = 50.45$, $p < 0.0001$; TimeBins: $F\ (22, 1892) = 45.15$, $p < 0.0001$; Interaction: $F\ (22, 1892) = 2.914$, $p < 0.0001$).

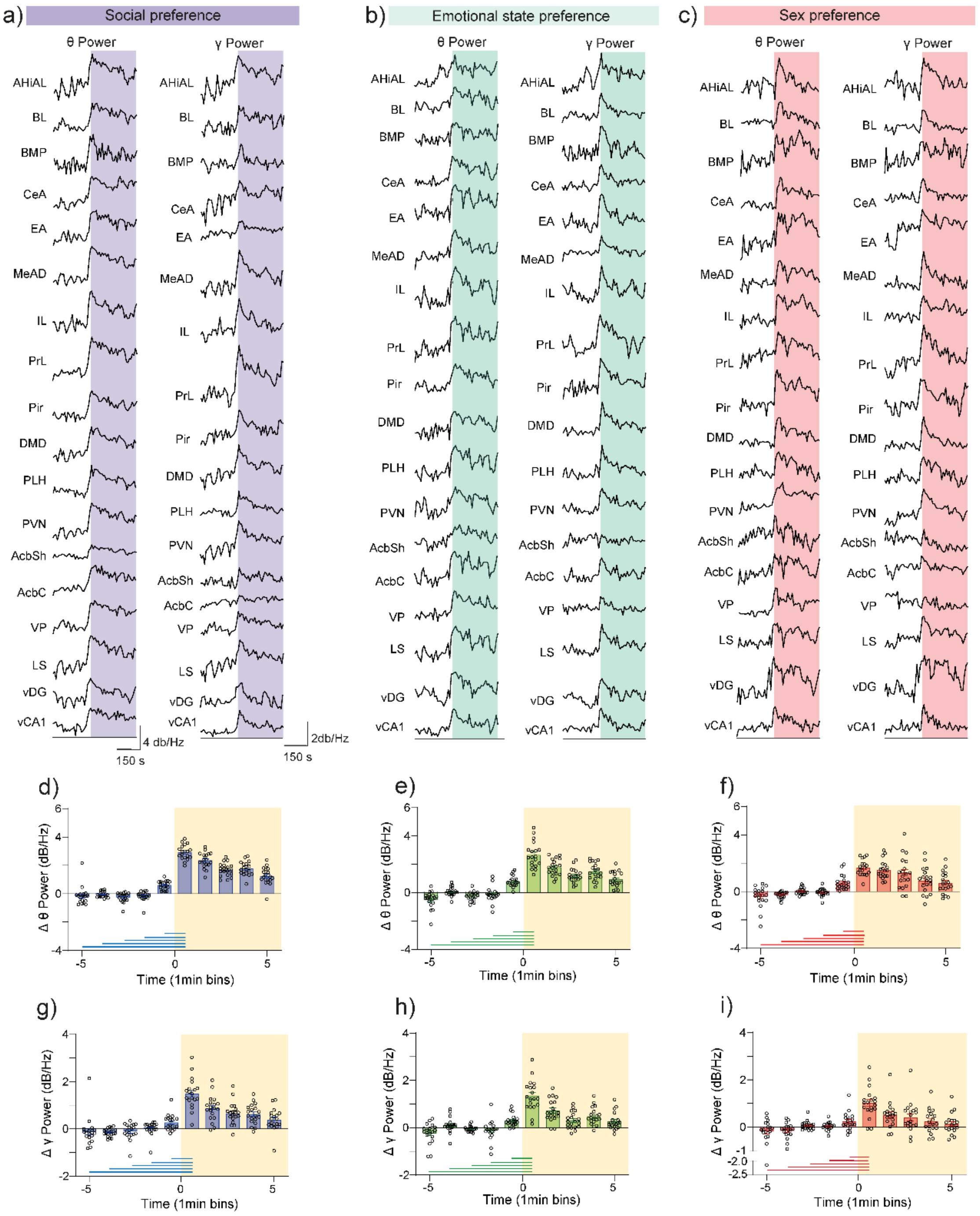

a)
Social preference
θ Power
γ Power
AHiAL
BL
BMP
CeA
EA
MeAD
IL
PrL
Pir
DMD
PLH
PVN
AcbSh
AcbC
VP
LS
vDG
vCA1
4 db/Hz
150 s
2db/Hz
150 s
b)
Emotional state preference
c)
Sex preference
d)
e)
f)
g)
h)
i)
Δ θ Power (dB/Hz)
Δ γ Power (dB/Hz)
Time (1min bins)

Supplementary Figure 2. Similar dynamics of theta and gamma power across the various tasks and brain regions

a) Mean traces of ΔθP (left column) and ΔγP (right column) across all sessions of the SP task for each brain region. The colored bar represents the encounter period.

b) As in **a**, for the EsP task

c) As in **a**, for the SxP task

d) Mean (±SEM) ΔθP, averaged across all brain regions for every minute of the baseline and encounters periods of the SP task. Time '0' represents the time of stimuli insertion. Lines below the bars represent a significant difference between the first minute of the encounter and every minute of the baseline period.

e) As in **d**, for the EsP task.

f) As in **d**, for the SxP task

g-i) As in **d-f**, for ΔγP.

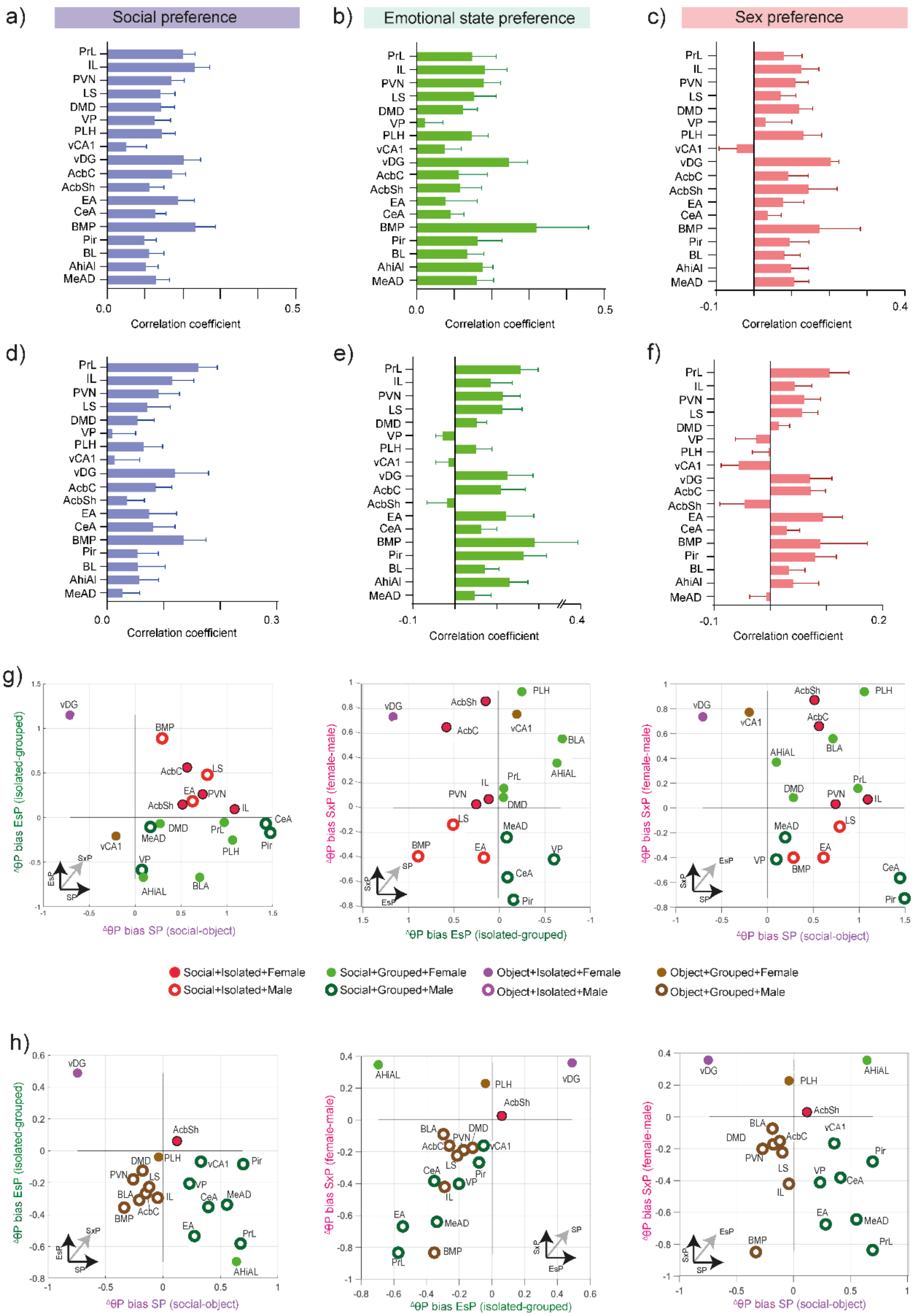

a) Social preference
b) Emotional state preference
c) Sex preference
PrL
IL
PVN
LS
DMD
VP
PLH
vCA1
vDG
AcbC
AcbSh
EA
CeA
BMP
Pir
BL
AhiAl
MeAD
Correlation coefficient
0.0
0.5
-0.1
0.4
d)
e)
f)
0.3
0.2
g)
h)
ΔθP bias EsP (isolated-grouped)
ΔθP bias SP (social-object)
ΔθP bias SxP (female-male)
SP
EsP
SxP
AHiAL
BLA
Social+Isolated+Female
Social+Isolated+Male
Social+Grouped+Female
Social+Grouped+Male
Object+Isolated+Female
Object+Isolated+Male
Object+Grouped+Female
Object+Grouped+Male

Supplementary Figure 3. Correlations between theta and gamma power changes during social encounters and subject speed and 2D maps of bias in $^{\Delta}\theta$P and $^{\Delta}\gamma$P across the various regions.

- **a)** Correlation coefficients across all SP task sessions for calculating Pearson's correlation between the change in theta power (ΔθP) and the mean speed of the subject.
- **b)** As in **a**, for EsP sessions.
- **c)** As in **a**, for SxP sessions.
- **d-f)** As in **a**-**c**, for gamma power (ΔγP).
- **g)** 2D plots of the mean difference in ΔθP between preferred and less-preferred stimuli, for the EsP task as a function of the SP task (left), SxP as a function of EsP (middle), and SxP as a function of SP (right). Each circle represents a given brain region, color- and shape-coded according to the combined bias across all tasks. See legend of the color and shape code of the distinct combinations below.
- **h)** As in **g**, for $^{\Delta}\gamma$P.

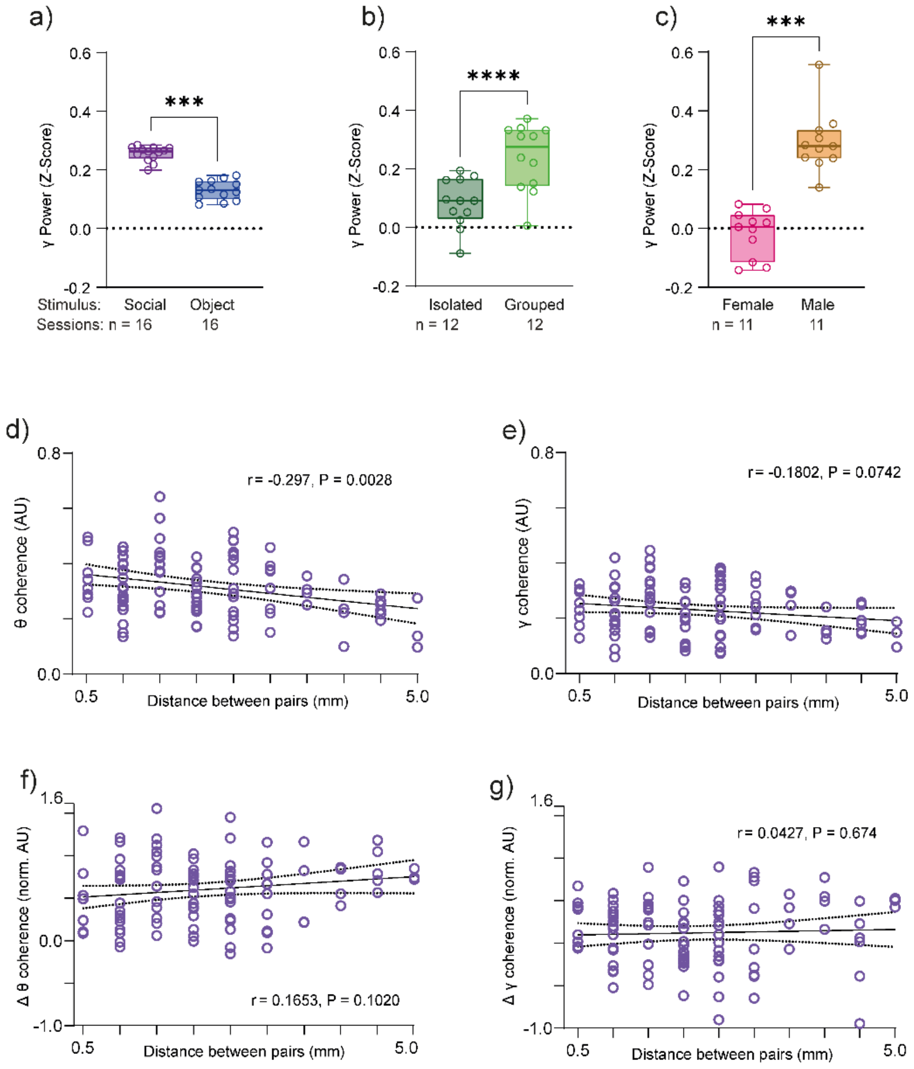

Supplementary Figure 4. Gamma power bias and volume conduction

a) Mean (±SEM) change in gamma power (relative to baseline) of LFP signals recorded in the extended amygdala during investigation bouts towards the social (left) and object (right) stimuli in the SP task, averaged over -1 to +1 s relative to begging of bout, across all subject. Stimulus type and sample size are denoted below. Wilcoxon matched pairs signed rank test, n = 15 sessions, W = -91, ***p = 0.002.

b) As in **a**, for the EsP task. Paired t-test: n = 12 sessions, t (11) = 8.379, ****p < 0.0001.

c) As in **a**, for the SxP task. Paired t-test: n = 11 sessions, t (10) = 6.099, ***p = 0.001.

d) Pearson correlation between the theta coherence during the baseline stage of the SP task (before introduction of stimuli), averaged across all subjects, and the distance between each two brain regions, across all pairs (n=99), with each circle representing one pair. Correlation coefficient and significance are written above the plot.

e) As in **d**, for gamma coherence.

f) As in **d**, for the change in theta coherence during the encounter, as compared to baseline, of the SP task.

g) As in **f**, for the change in gamma coherence.

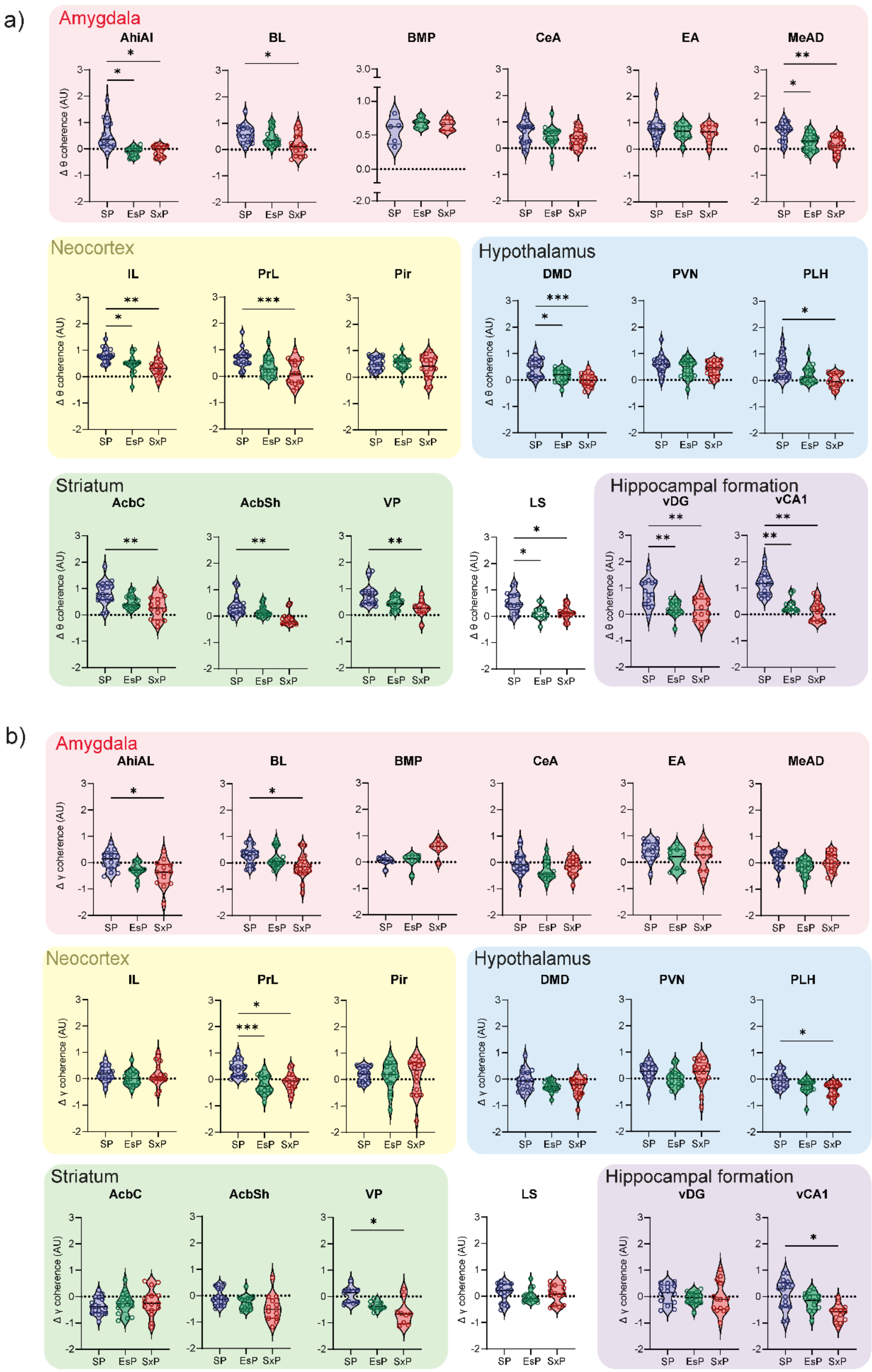

a)
Amygdala
AhiAl
BL
BMP
CeA
EA
MeAD
Δ θ coherence (AU)
SP
EsP
SxP
Neocortex
IL
PrL
Pir
Hypothalamus
DMD
PVN
PLH
Striatum
AcbC
AcbSh
VP
LS
Hippocampal formation
vDG
vCA1
b)
Amygdala
AhiAL
BL
BMP
CeA
EA
MeAD
Δ γ coherence (AU)
Neocortex
IL
PrL
Pir
Hypothalamus
DMD
PVN
PLH
Striatum
AcbC
AcbSh
VP
LS
Hippocampal formation
vDG
vCA1

Supplementary Figure 5. Coherence changes during the encounter period of the various tasks are brain region-specific

a) Mean change in theta coherence (ΔθCo) between a given brain region and all other simultaneously recorded regions during the encounter period, compared across the three tasks. The various regions are grouped together according to their location in the brain by colored background.

b) As in **a**, for gamma coherence (ΔγCo).

*$p < 0.03$, **$p < 0.002$, ***$p = 0.0002$, ****$p < 0.0001$, Dunnett's and Dunn's *post-hoc* test after FDR correction, following the main effect in ANOVA and Kruskal-Wallis test, respectively.

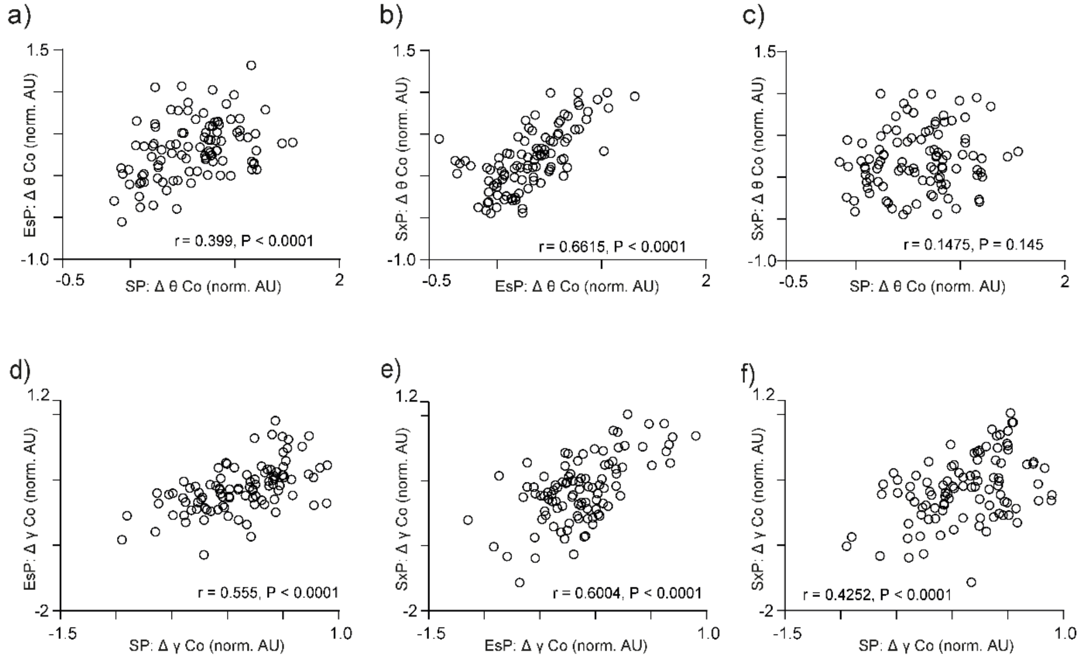

Figure S6. Correlations in theta and gamma coherence changes during social encounter between tasks

a) A correlation between theta coherence change ($^{\Delta}\theta$Co) during the encounter periods of the SP and EsP tasks, across all 99 couples of brain regions. The coefficient and significance of the correlation are denoted below.

b) As in **a**, for the correlation between EsP and SxP. Note the especially strong correlation between these two tasks.

c) As in **a**, for the correlation between SP and SxP. Note the lack of correlation between these two tasks.

d-f) As in **a-c**, for gamma coherence change ($\Delta\gamma$Co).

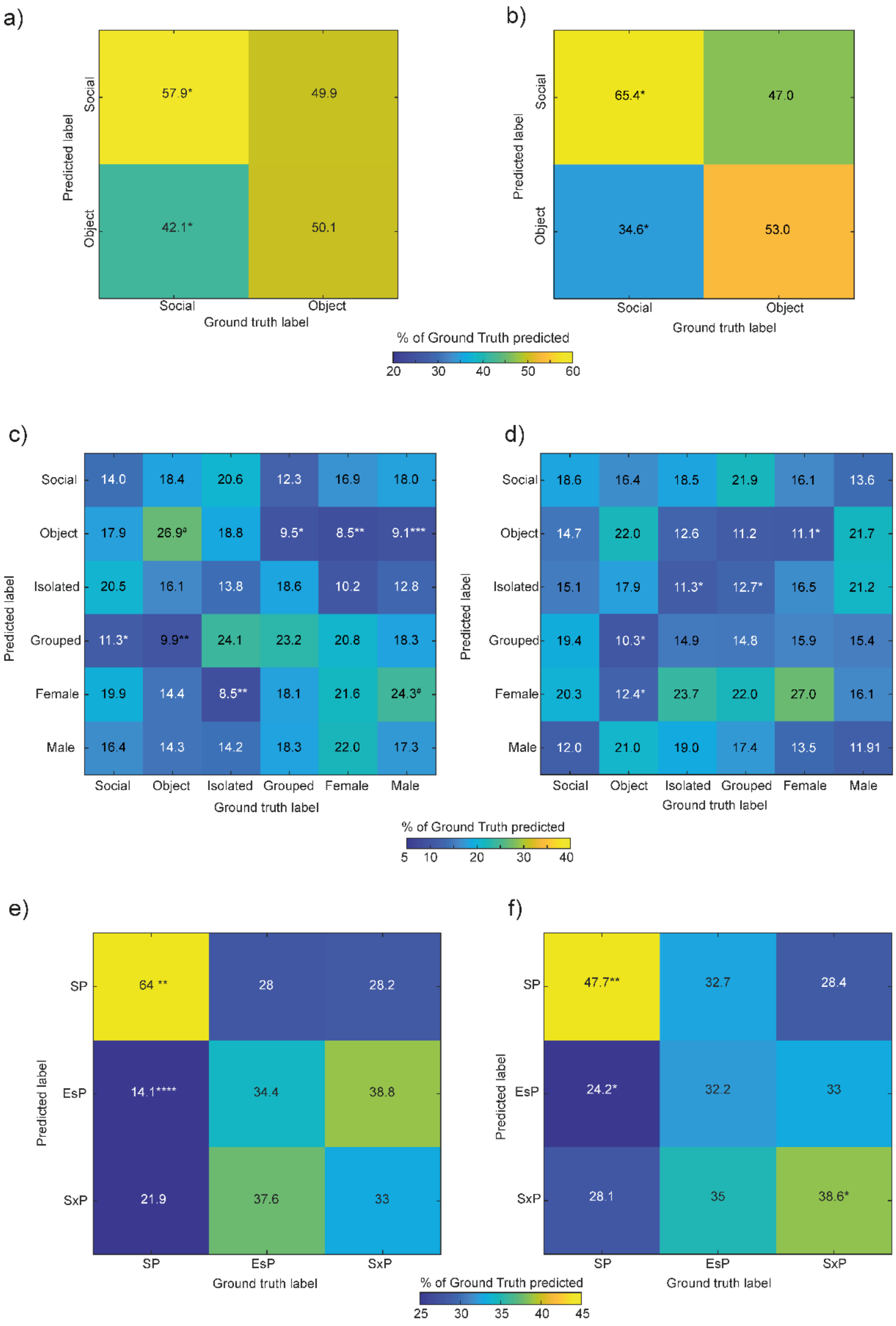

a)
Predicted label
Social
Object
57.9*
49.9
42.1*
50.1
Social
Object
Ground truth label
b)
Predicted label
Social
Object
65.4*
47.0
34.6*
53.0
Social
Object
Ground truth label
% of Ground Truth predicted
20 30 40 50 60
c)
Predicted label
Social 14.0 18.4 20.6 12.3 16.9 18.0
Object 17.9 26.9# 18.8 9.5* 8.5** 9.1***
Isolated 20.5 16.1 13.8 18.6 10.2 12.8
Grouped 11.3* 9.9** 24.1 23.2 20.8 18.3
Female 19.9 14.4 8.5** 18.1 21.6 24.3#
Male 16.4 14.3 14.2 18.3 22.0 17.3
Social Object Isolated Grouped Female Male
Ground truth label
d)
Predicted label
Social 18.6 16.4 18.5 21.9 16.1 13.6
Object 14.7 22.0 12.6 11.2 11.1* 21.7
Isolated 15.1 17.9 11.3* 12.7* 16.5 21.2
Grouped 19.4 10.3* 14.9 14.8 15.9 15.4
Female 20.3 12.4* 23.7 22.0 27.0 16.1
Male 12.0 21.0 19.0 17.4 13.5 11.91
Social Object Isolated Grouped Female Male
Ground truth label
% of Ground Truth predicted
5 10 20 30 40
e)
Predicted label
SP 64 ** 28 28.2
EsP 14.1**** 34.4 38.8
SxP 21.9 37.6 33
SP EsP SxP
Ground truth label
f)
Predicted label
SP 47.7** 32.7 28.4
EsP 24.2* 32.2 33
SxP 28.1 35 38.6*
SP EsP SxP
Ground truth label
% of Ground Truth predicted
25 30 35 40 45

Supplementary Figure 7. Random forest model can predict the social stimulus in the SP task but not the specific stimulus among all six stimuli

a) A color-coded confusion matrix of a multi-class Random forest classifier using the changes in theta coherence during investigation bouts ($^{\Delta}\theta$Co) across the SP task to predict the type of investigated stimulus among the two possible stimuli (social and object).

b) As in **a**, for gamma coherence ($^{\Delta}\gamma$Co).

c) A color-coded confusion matrix of the same model using $^{\Delta}\theta$Co across all tasks to predict the type of investigated stimulus among the six possible stimuli.

d) As in **c**, for $^{\Delta}\gamma$Co.

e) A color-coded confusion matrix for the Random Forest classifier employed for predicting the social context from $^{\Delta}\theta$P values across all brain regions and stimuli. The scale of the accuracy's color code is shown to the right. The percentage of cases a label was predicted for each ground truth are marked in the middle of each spot. *$p < 0.05$, **$p < 0.01$, ***$p = 0.0001$, ****$p < 0.0001$, Mann-Whitney test, FDR corrected.

f) As in **e**, for the combination of $^{\Delta}\theta$P and $^{\Delta}\theta$Co. *$p < 0.05$, **$p < 0.01$, Mann-Whitney test, FDR corrected.

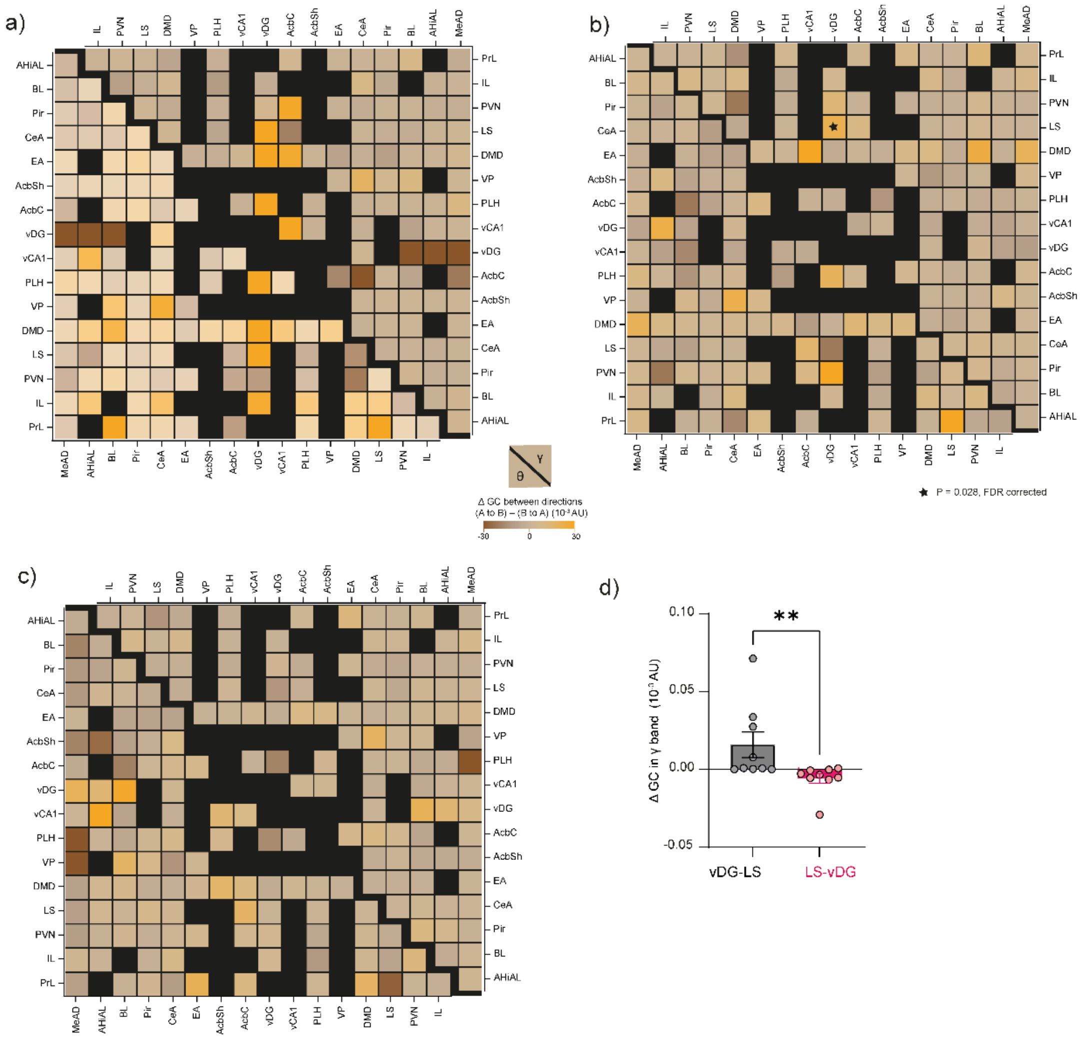

Supplementary Figure 8. Directionality of granger causality (GC) across coupled brain regions and tasks

- **a)** Color-coded matrices across all couples of recorded brain regions, of differences in GC changes (relative to baseline) during the SP task, between the two directions (region A to regions B and vice versa), for the theta (lower left) and gamma (upper right) bands.
- **b)** As in **a**, for the EsP task. **p* < 0.05, Mann-Whitney test, FDR corrected.
- **c)** As in **a**, for the SxP task.
- **d)** Mean (±SEM) change in gamma GC from vDG to LS (Grey) and from LS to vDG pink. Wilcoxon matched pairs signed rank test, n = 9 sessions, W = -43, ***P* = 0.0078.

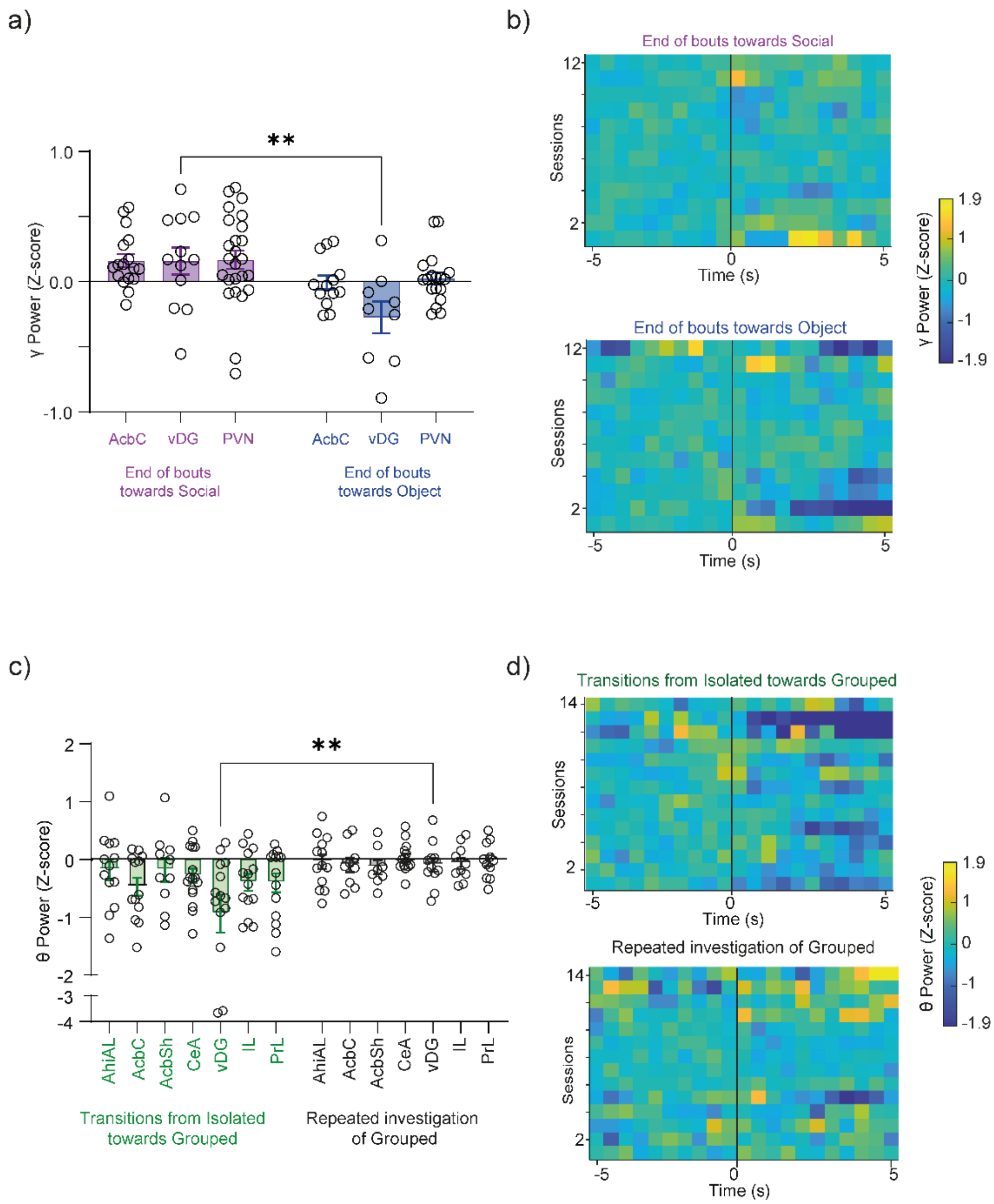

Supplementary Figure 9. Significant difference in gamma and theta power for specific behavioral events

a) Mean (±SEM) Z-score values of gamma power change in the vDG at the end of investigation bouts towards social (purple) and object (blue) stimuli during SP task sessions, shown for three brain regions where significant changes in GC were found in the gamma band. The mean was calculated across five seconds following the end of bout (Mixed-effects model (REML)). Areas x Stimulus, Areas: F (2, 88) = 2.111, *p*=0.1273; Stimulus: F (1, 88) = 15.69, *p* = 0.0002; Interaction: F (2, 88) = 1.898, *p* = 0.1559, Šídák's post hoc test ***p* = 0.0029).

b) Heat maps of vDG gamma power changes across five seconds before and five seconds after the beginning of the end of investigation bouts towards social (above) and object (below)

stimuli during SP task sessions. Each row represents the mean Z-score of all bouts in a single session (bins of 0.5 s). Time point **'0'** represents the end of the bout. The color code is on the right.

c) As in **a**, for vDG Theta power during transitions in EsP task (Mixed-effects model (REML)). Areas x Stimulus, Areas: F (6, 87) = 1.459, p = 0.2017; Stimulus: F (1, 69) = 14.81, p = 0.0003; Interaction: F (6, 69) = 1.072, p = 0.3875; Šídák's post hoc test **p=0.007).

d) Heat maps of vDG theta power changes across five seconds before and five seconds after the beginning of investigation bouts after transitions from the isolated to grouped stimulus (above); and repeated investigations of grouped stimuli (below) across all EsP task sessions.
