# Supplementary material for: Synchronized LFP rhythmicity in the social brain reflects the context of social encounters
Source: Commun Biol. 2024 Jan 2;7:2. doi: 10.1038/s42003-023-05728-8 (PMC10761981; doi:10.1038/s42003-023-05728-8)
Supplement: Supplementary file 3 — Description of Additional Supplementary Files [file 42003_2023_5728_MOESM3_ESM.pdf]

Description of Supplementary Data files

File name: Supplementary Data 1

Description: The source data behind all the graphs in the paper.

File name: Supplementary Data 2

Description: A list of all sessions and all targeted and miss-targeted brain regions of each subject mouse.

File name: Supplementary Data 3

Description: Statistical summary of synchronization to each behavioral event for all tasks and LFP rhythms.

File name: Supplementary Data 4

Description: Summary of the details and results of all statistical tests used in the paper.
